# A case report of Hirschsprung’s disease presenting as sigmoid volvulus and literature review, Tikur Anbessa Specialized Hospital, Addis Ababa, Ethiopia

**DOI:** 10.1186/s12893-020-00938-x

**Published:** 2021-03-03

**Authors:** Abay Wondimu Gosaye, Temesgen Setato Nane, Tihitena Mammo Negussie

**Affiliations:** Department of Surgery, Tikur Anbessa Specialized Hospital, Addis Ababa University, Zambia St., Addis Ababa, Ethiopia

**Keywords:** Hirschsprung’s disease, Sigmoid volvulus, Pediatrics

## Abstract

**Background:**

Sigmoid volvulus is an uncommon problem in children and adolescents, and is rarely considered a diagnosis in this group. A high index of suspicion is necessary to reach a diagnosis and avoid morbidity and mortality. Sigmoid volvulus is a rare complication of Hirschsprung’s disease, which has been reported in neonates, children, and adults. Here we report a case of sigmoid volvulus accompanied by undiagnosed Hirschsprung's disease.

**Case presentation:**

A 9 years old boy who presented with sudden onset of colicky abdominal pain of 4 h duration associated with gross abdominal distension and 2 episodes of non-bilious vomiting. A plain abdominal radiographs showed single hugely dilated bowel loops in the left lower quadrant with single air fluid level. Sigmoid volvulus was considered and rectal tube deflation was done and it was successful. Full thickness rectal biopsy was done and it was consistent with aganglionic megacolon. A primary trans-anal Soave endo-rectal pull through was done 3 weeks later, after biopsy result arrived, which yielded loss of symptoms and regular bowel movement.

**Conclusions:**

Sigmoid volvulus should be considered in the differential for children presenting with acute onset of abdominal obstruction. It should be known that when its’s diagnosed in children, it is often associated with Hirschsprung's disease. Therefore, a proper diagnostic and treatment algorithm should be followed in order not to miss associated HD and to give optimum care to such patients.

## Background

Sigmoid volvulus is an uncommon problem in children and adolescents, and is rarely considered a diagnosis in this group [1]. A high index of suspicion is necessary to reach a diagnosis and avoid morbidity and mortality. Hirschsprung’s disease has been implicated in sigmoid volvulus [2]. Although Hirschsprung’s disease is most often diagnosed in the neonatal period, it can also present during late infancy, early and late childhood, and even adulthood. Sigmoid volvulus is a rare complication of Hirschsprung’s disease, which has been reported in neonates, children, and adults [3–9]. This report describes a 9 years old male who presented with sudden onset colicky abdominal pain, failure to pass feces and flatus and abdominal distension and was found to have sigmoid volvulus with previously unrecognized Hirschsprung’s disease.

## Case presentation

A 9 years old, previously healthy boy presented with sudden onset of colicky abdominal pain of 4 h duration. The pain was mainly infra-umbilical. Associated with this he had gross abdominal distension and 2 episodes of non-bilious vomiting. Parents reported that the child had bowel movements daily before he joined school since when bowel movement became every 2–3 days. The parents do not remember when the child passed the first meconium. The child had had similar attack a month before presentation to our hospital and rectal tube deflation was done at a nearby hospital. Otherwise there was no other complaints. His growth and development is similar compared to his peers. He was not on any medications. There was no family history of similar illnesses.

**Fig. 1:**
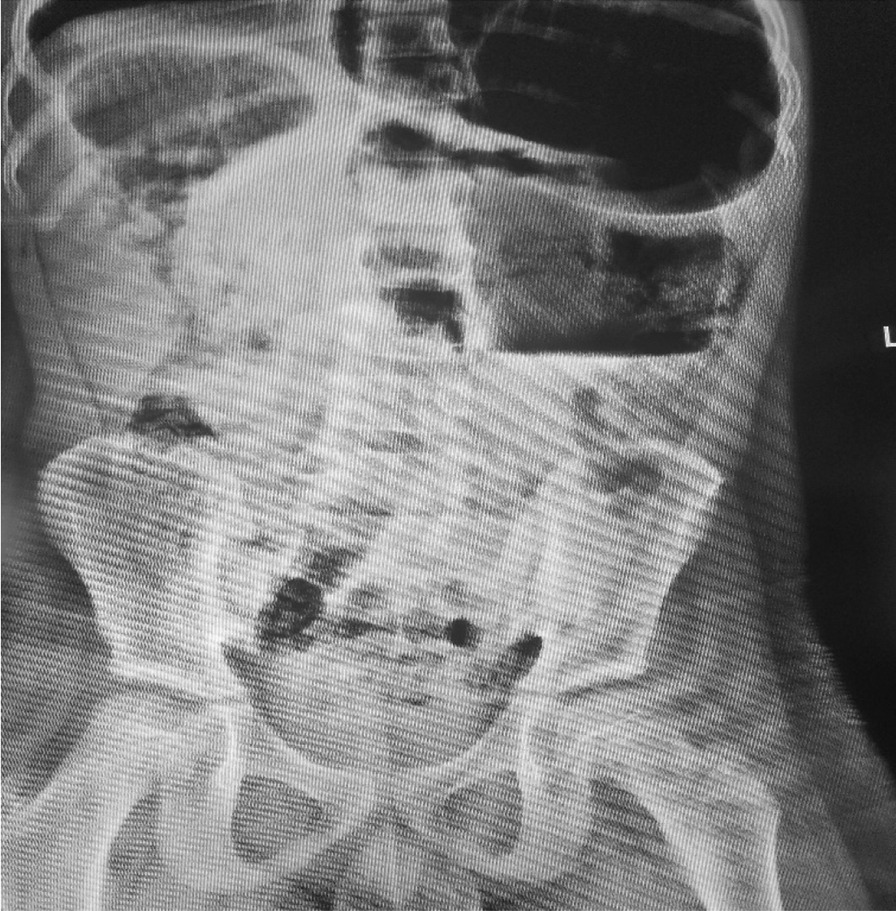
PA plain abdominal x-ray showing single air fluid level with coffee bean appearance

On physical examination except for tachycardia other vital signs were all in normal range. All anthropometric measurements were in normal range. He was in pain while holding his abdomen. He had gross abdominal distension. There was no direct or rebound tenderness. Abdomen was hyper tympanic to percussion. Bowel sounds were increased. There was no organomegally or palpable masses on deep palpation. Per-rectum examination showed empty rectum. No stool or blood on examining finger. A Plain abdominal radiographs showed single hugely dilated bowel loops in the left lower quadrant with single air fluid level (Fig. 1). Abdominal ultrasound was unremarkable except for gas filled abdomen. Complete blood count and organ function test was done and all were in normal range.

**Fig. 2:**
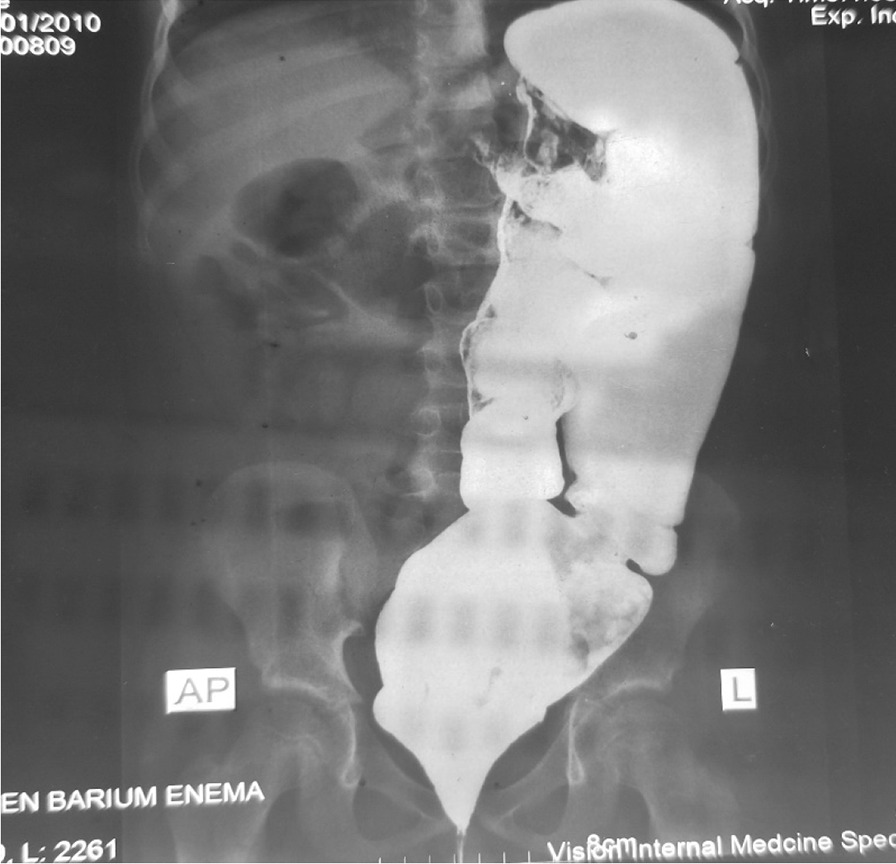
PA barium enema study showing dilated and redundant sigmoid colon without radiologic evidence of Hirschsprung’s disease

**Fig. 3:**
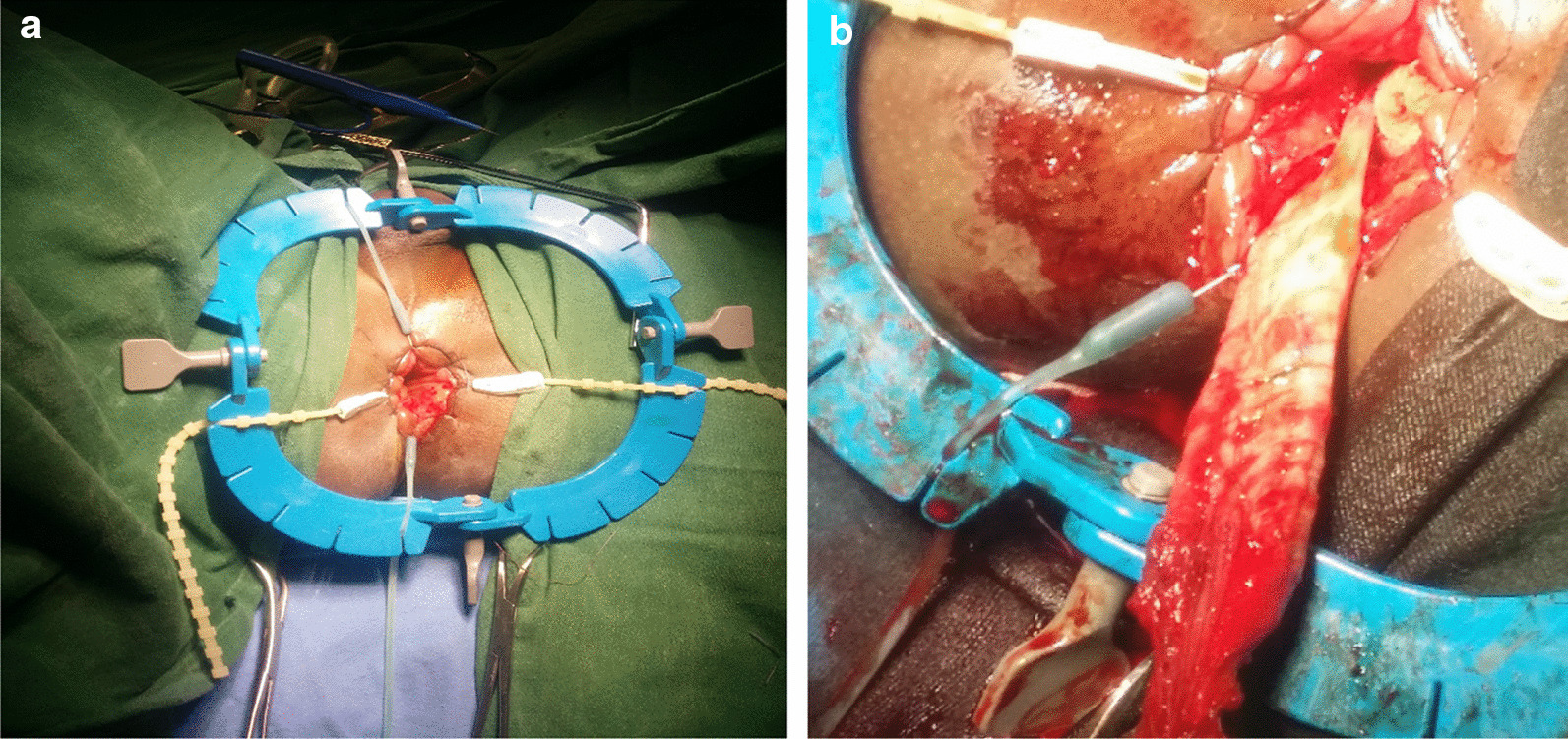
**a**,** b** Pictures showing trans-anal Soave’s endo-rectal pull through

We put the patient in knee-chest position and gently introduced fully lubricated rectal tube into the rectum and slowed advanced upward. Gush of air and scanty stool came out. The abdomen became soft and the cramp subsided. We left the tube in the rectum for 24 h. We took full thickness rectal biopsy the next day. It showed aganglionated rectum with absent ganglion cell in both submucosal and muscles layers. We also obtained diagnostic barium enema and it showed redundant sigmoid with no radiologic evidence of Hirschsprung’s disease (Fig. 2). After Hirschsprung’s disease confirmed by rectal biopsy, he underwent primary trans-anal Soave’s endorectal pull through (Fig. 3). We resected the rectum and dilated part of sigmoid colon until normal appearing bowel caliber is reached. We did colo-rectal end-to-end anastomosis 2 cm above dentate line. He was kept nothing per-os for the 48 h post-op and was put on prophylactic antibiotics.

He was then started on feeding and tolerated it and discharged on 6th post-op day. He experienced passage of loose frequent stool for the first 10 days post-operatively which improved later by itself.

Currently, on his third post-operative months, he is having regular bowel movement. Result of the excision biopsy showed aganglionated distal colon and ganglionated proximal bowel.

## Discussion and conclusions

Sigmoid volvulus is a condition in which the sigmoid colon rotates around its elongated mesentery, resulting in a closed-loop obstruction. This obstruction of the intestinal lumen and impairment of vascular perfusion occur when the degree of torsion exceeds 180 and 360, respectively [10]. Sigmoid volvulus is rarely seen in patients with HD, with a reported prevalence of 0.66% [3]. Conversely, among children presenting with SV, 18% also had HD [3, 8]. In pediatrics age group, the median age of diagnosis is approximately 7–12 years old with male to female ratio of 3.5:1, but one case series in Turkey showed that 17 out of 19 cases were male making male to female 9:1 [9, 11].

Children with SV associated with HD are reported to have a higher prevalence of the acute presentation (91% or 10/11) than those with SV and no HD (55% or 28/51) [9]. Prompt diagnosis of the acute presentation of volvulus has been found to decrease morbidity and increase survival [11, 12]. In adults, abdominal radiographs identify the omega or coffee bean sign in more than 60% of cases, but in children this has only a 17–30% success rate [9, 13, 14]. The most common findings in radiographs of children are colonic distention or an air-fluid level in sigmoid loops [1, 9].

**Fig. 4:**
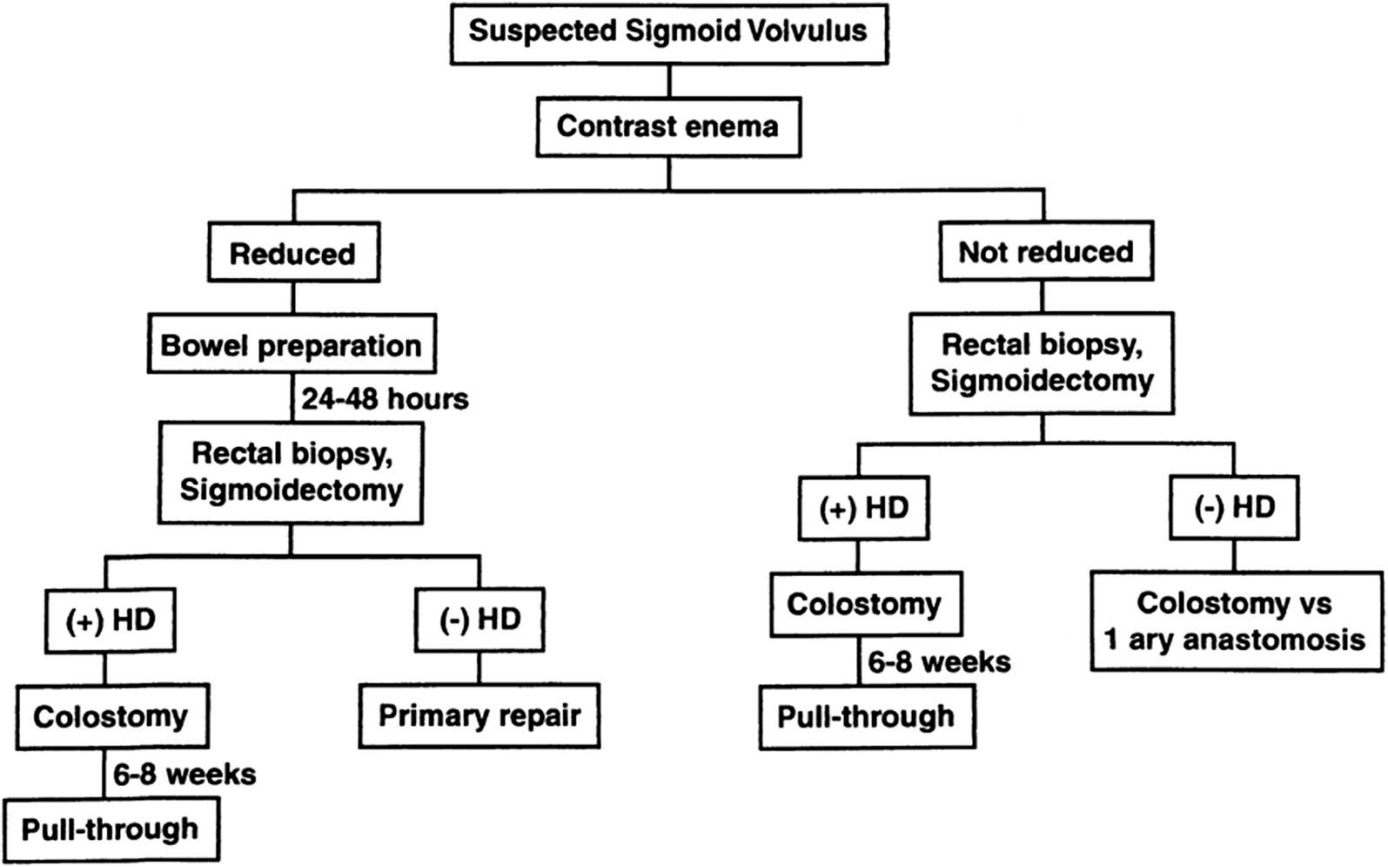
Algorithm for the management of sigmoid volvulus in children and adolescents proposed by Salas et al. HD, Hirschsprung’s Disease

**Fig. 5:**
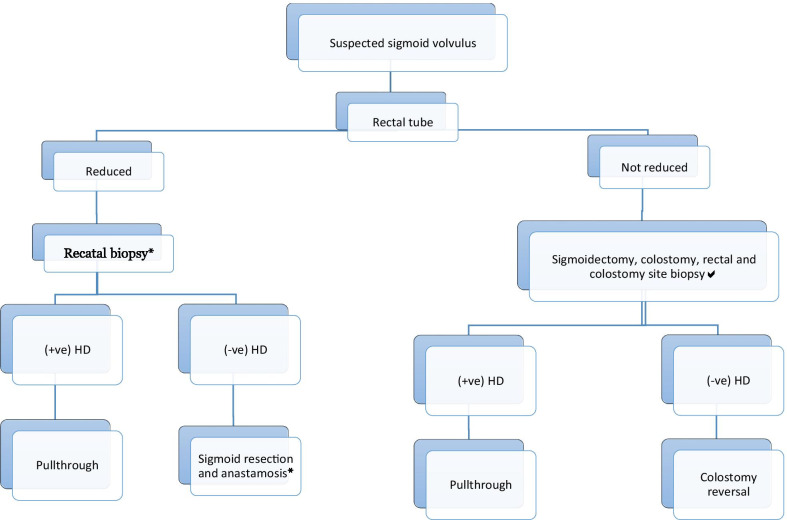
Modified Salas’s et al. algorithm for the management of sigmoid volvulus in children and adolescents proposed by Abay et al. for low income countries. *HD* Hirschsprung’s disease. *Sigmoid resection and anastomosis should be done on semi-emergency basis as soon as rectal biopsy result arrives

Evidences have shown that there is association between sigmoid volvulus and HD, and a full-thickness biopsy is required to rule out aganglionosis [3, 10, 14, 15]. Therefore, we propose a modified algorithm to the one suggested by Salas et al. (Fig. 5) to adopt in low income countries where barium enema, colonoscopy and frozen section is not readily available. Original algorithm developed by Salas and his colleagues requires frozen section service and expertise (Fig. 4). Rectal tube can be used in acute non-gangrenous sigmoid volvulus for deflation. If deflation is successful rectal tube should be kept in place for 24–48 h and rectal biopsy should be taken in the same admission. Based on biopsy result pull though/sigmoidectomy should be done urgently as soon as result is arrived because of high risk of early recurrence rates [9, 16]. This is even more important in pediatrics age group, because of the patients’ longer average life expectancy [9, 14, 16, 17].

When tube deflation is not successful or the patient develops signs of perforation/peritonitis emergency laparotomy is warranted [12, 18]. The definitive treatment being sigmoidectomy, either with primary anastomosis or colostomy [19]. But studies have shown that resection with primary anastomosis in emergency situations, when the general condition of the patient is suboptimal and the bowel is not prepared, carries an unacceptably high complication rate [20], thus we suggest to avoid primary anastomosis. Sigmoidectomy, colostomy and rectal and colostomy site biopsy, in a setting like ours where we don’t have frozen section, should be done to know presence and extend of aganglionosis. Pull through or colostomy reversal will be done after 6–8 weeks based on the result of biopsy (Fig. 5).

Sigmoid volvulus should be considered in the differential for children presenting with acute onset of abdominal obstruction. It should be known that when its’s diagnosed in children, it is often associated with Hirschsprung's disease. Therefore, a proper diagnostic and treatment algorithm should be followed in order not to miss associated HD and to give optimum care to such patients.

### Limitations

As this is a single case report, information on epidemiological quantities cannot be generated. This is a very rare and atypical presentation of Hirshsprung disease and the findings from this case report cannot be generalized.

## Data Availability

The datasets used and/or analysed during the current study are available from the corresponding author on reasonable request.

## Abbreviations

HD: Hirshsprung’s disease
SV: Sigmoid volvulus
